# Supplementary material for: ATP synthesis of Enterococcus hirae V-ATPase driven by sodium motive force
Source: J Biol Chem. 2025 Mar 19;301(4):108422. doi: 10.1016/j.jbc.2025.108422 (PMC12018189; doi:10.1016/j.jbc.2025.108422)
Supplement: 250301_ATPsyn_SI_rev_RI_woRed.pdf [file mmc1.pdf]

**Supporting Information**

# ATP synthesis of *Enterococcus hirae* V-ATPase driven by sodium motive force

*Akihiro Otomo*[1,2*†], *Lucy Gao Hui Zhu*[3], *Yasuko Okuni*[1], *Mayuko Yamamoto*[1], *Ryota Iino*[1,2*]

[1] *Institute for Molecular Sciences, National Institutes of Natural Sciences, Okazaki, Aichi 444-8787, Japan*

[2] *Graduate Institute for Advanced Studies, SOKENDAI, Hayama, Kanazawa 240-0193, Japan*

[3] *Chimie Paris Tech, Paris 75231, France*

[†] Present address: *Department of Chemistry, Graduate School of Science, Kyoto University, Kitashirakawa-Oiwakecho, Sakyo-ku, Kyoto 606–8502, Japan*

**Table S1**. Concentrations of key components for the measurements in Figure 2 and Figure S3

| | [ADP] dependence | [Pi] dependence |
|---|---|---|
| $[Na^+]_{in}$ (mM) | 200 | 200 |
| $[Na^+]_{out}$ (mM) | 2.3 | 2.3 |
| $\Delta pNa$ (mV) | 114.6 | 114.6 |
| $[K^+]_{in}$ (mM) | 1.1 | 1.1 |
| $[K^+]_{out}$ (mM) | 454.4 | 454.4 |
| $\Delta\psi$ (mV) | 154.7 | 154.7 |
| ATP (nM) | 0* | 0* |
| ADP (mM) | 0.001 - 0.5 | 0.5 |
| Pi (mM) | 74 | 0.2 - 90 |

*ATP was not intentionally added but contaminated in ADP (<0.003%).

**Table S2**. Concentrations of key components for the measurements in Figure 3 and Figure S4

| | $\Delta\psi$ dependence | | | | | | |
|---|---|---|---|---|---|---|---|
| $[Na^+]_{in}$ (mM) | 250 | 250 | 250 | 250 | 250 | 250 | 250 |
| $[Na^+]_{out}$ (mM) | 12.0 | 12.5 | 12.5 | 12.0 | 12.0 | 12.0 | 12.0 |
| $\Delta$pNa (mV) | 78.0 | 77.0 | 77.0 | 78.0 | 78.0 | 78.0 | 78.0 |
| $[K^+]_{in}$ (mM) | 6.2 | 12.9 | 12.9 | 6.2 | 6.2 | 6.2 | 6.2 |
| $[K^+]_{out}$ (mM) | 45.1 | 40.6 | 63.2 | 45.1 | 58.6 | 76.7 | 96.6 |
| $\Delta\psi$ (mV) | 0* | 29.4 | 40.8 | 51.0 | 57.7 | 64.6 | 70.5 |
| ATP (nM) | 0** | | | | | | |
| ADP (mM) | 0.5 | | | | | | |
| Pi (mM) | 25 | | | | | | |

| | $\Delta$pNa dependence | | | | | | |
|---|---|---|---|---|---|---|---|
| $[Na^+]_{in}$ (mM) | 250 | 250 | 250 | 250 | 250 | 250 | 250 |
| $[Na^+]_{out}$ (mM) | 250.7 | 78.9 | 50.9 | 33.7 | 26.5 | 20.2 | 15.6 |
| $\Delta$pNa (mV) | 0 | 29.5 | 40.9 | 51.5 | 57.7 | 64.7 | 71.2 |
| $[K^+]_{in}$ (mM) | 6.2 | 12.9 | 12.9 | 6.2 | 6.2 | 6.2 | 6.2 |
| $[K^+]_{in}$ (mM) | 122 | 257.6 | 257.6 | 122 | 122 | 122 | 122 |
| $\Delta\psi$ (mV) | 76.5 | 76.9 | 76.9 | 76.5 | 76.5 | 76.5 | 76.5 |
| ATP (nM) | 0** | | | | | | |
| ADP (mM) | 0.5 | | | | | | |
| Pi (mM) | 25 | | | | | | |

*Valinomycin was not added so $\Delta\psi$ is practically zero.

**ATP was not intentionally added but contaminated in ADP (<0.003%).

**Table S3**. Concentrations of key components for the measurements in Figure 4 and Figure S5

| | | | | | | | |
|---|---|---|---|---|---|---|---|
| $[Na^+]_{in}$ (mM) | 250 | 250 | 250 | 250 | 250 | 250 | 250 |
| $[Na^+]_{out}$ (mM) | 20.2 | 20.2 | 20.2 | 20.2 | 20.2 | 20.2 | 20.2 |
| $\Delta$pNa (mV) | 64.6 | 64.6 | 64.6 | 64.6 | 64.6 | 64.6 | 64.6 |
| $[K^+]_{in}$ (mM) | 6.2 | 6.2 | 6.2 | 6.2 | 6.2 | 6.2 | 6.2 |
| $[K^+]_{out}$ (mM) | 19.1 | 28.2 | 37.4 | 46.5 | 55.6 | 64.7 | 87.5 |
| $\Delta\psi$ (mV) | 28.9 | 38.9 | 46.2 | 51.8 | 56.4 | 60.2 | 68.0 |
| ATP (nM) | 25 | | | | | | |
| ADP (mM) | 0.01 - 0.08 | | | | | | |
| Pi (mM) | 9.95 | | | | | | |

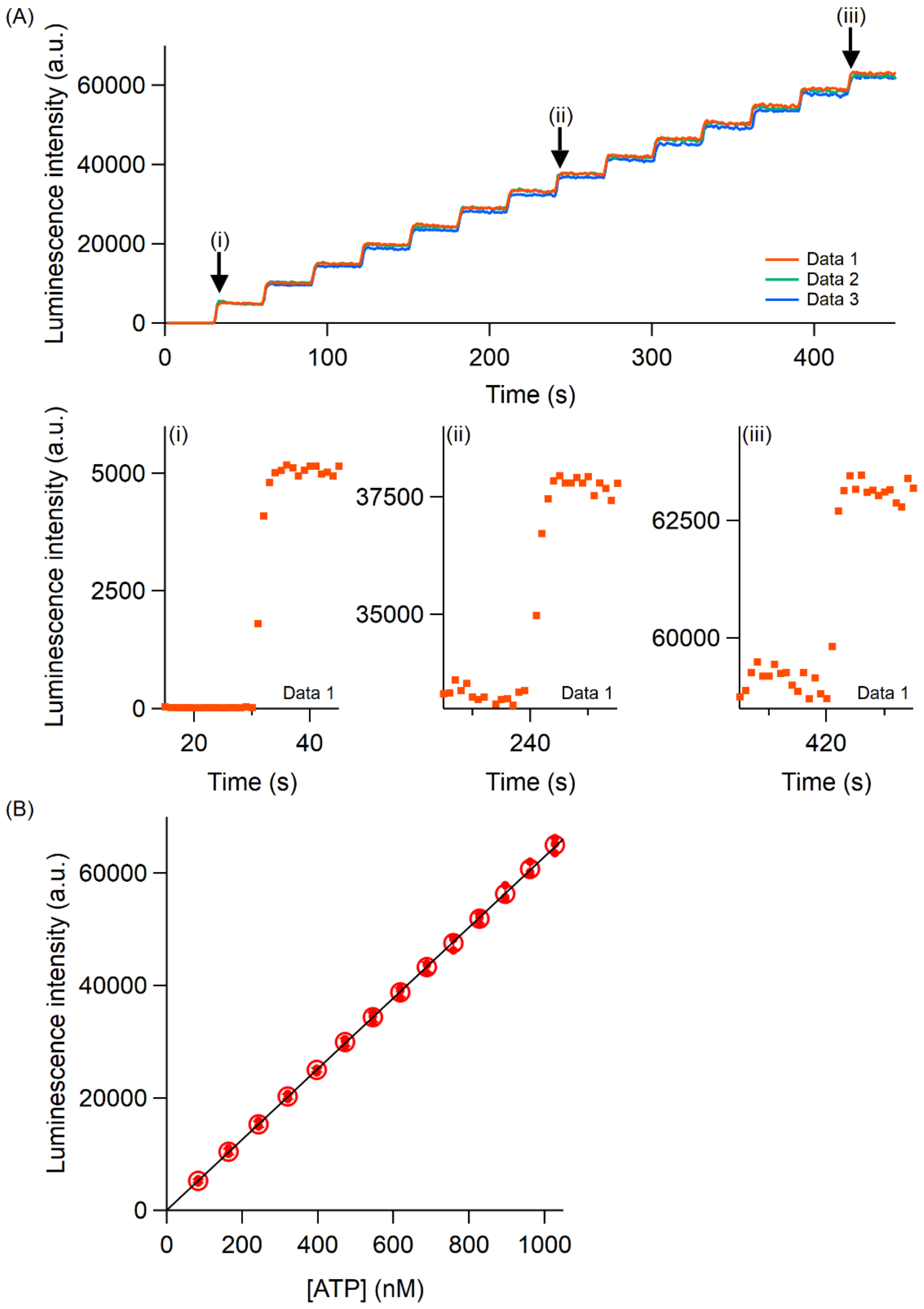


**Figure S1**. [ATP] dependence of luminescence intensity using luciferin/luciferase system. (A) Time courses of luminescence intensity upon sequential additions of ATP (4.2 μM, 20 μL each) at 30-second intervals to 1 mL of luciferin/luciferase solution. Three independent traces (Data 1, 2, and 3) are shown. Arrows (i, ii, and iii) indicate three selected intensity jumps following ATP additions in Data 1, with their magnified views shown at the bottom. (B) Correlation between [ATP] and luminescence intensity. Individual data points are shown as filled red circles and open red circles represent mean values with error bars indicating standard deviations (N = 3). The black line represents linear fit to data ($R^2 = 0.99$).

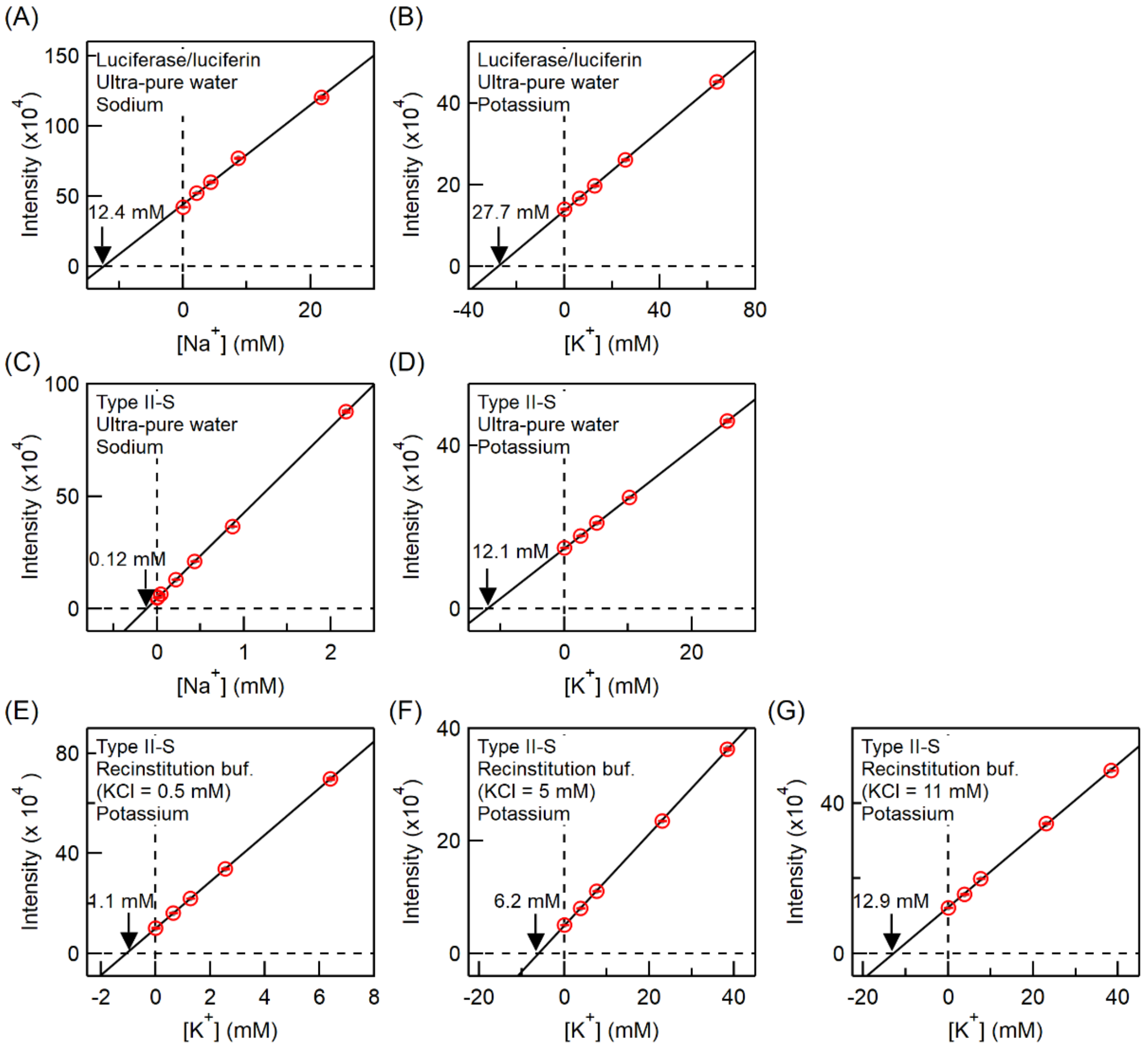


**Figure S2**. Quantitative analysis of $Na^+$ and $K^+$ in luciferin/luciferase reagent and Type II-S lipid by ICP-OES using standard addition method. The X-intercepts (indicated by black arrows) obtained by extrapolation of the calibration curves give the concentration of analyte in the samples. (A and B) $Na^+$ and $K^+$ concentrations in 18 mg/mL of the luciferin/luciferase reagent dissolved in ultra-pure water. (C and D) $Na^+$ and $K^+$ concentrations in 40 mg/mL of the Type II-S lipid suspended in ultra-pure water. (E to G) $K^+$ concentrations in 40 mg/mL of Type II-S lipids suspended in reconstitution buffers prepared with 0.5, 5 mM, and 11 mM of KCl, respectively.

(A)

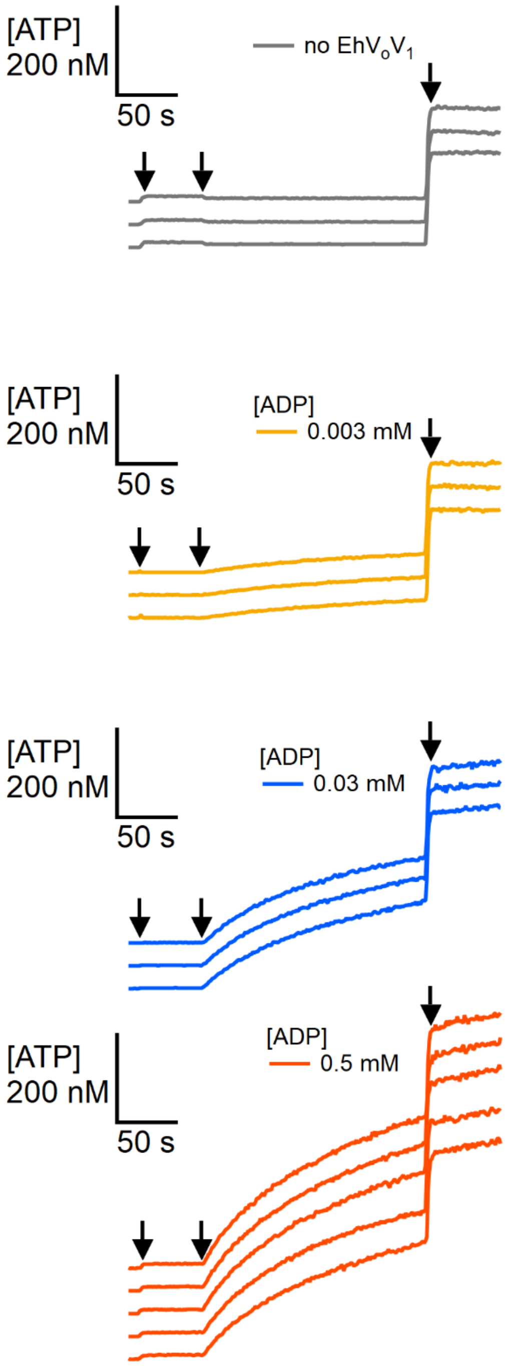

[ATP] 200 nM
50 s
no EhVoV1
[ATP] 200 nM
50 s
[ADP] 0.001 mM
[ATP] 200 nM
50 s
[ADP] 0.003 mM
[ATP] 200 nM
50 s
[ADP] 0.01 mM
[ATP] 200 nM
50 s
[ADP] 0.03 mM
[ATP] 200 nM
50 s
[ADP] 0.1 mM
[ATP] 200 nM
50 s
[ADP] 0.5 mM

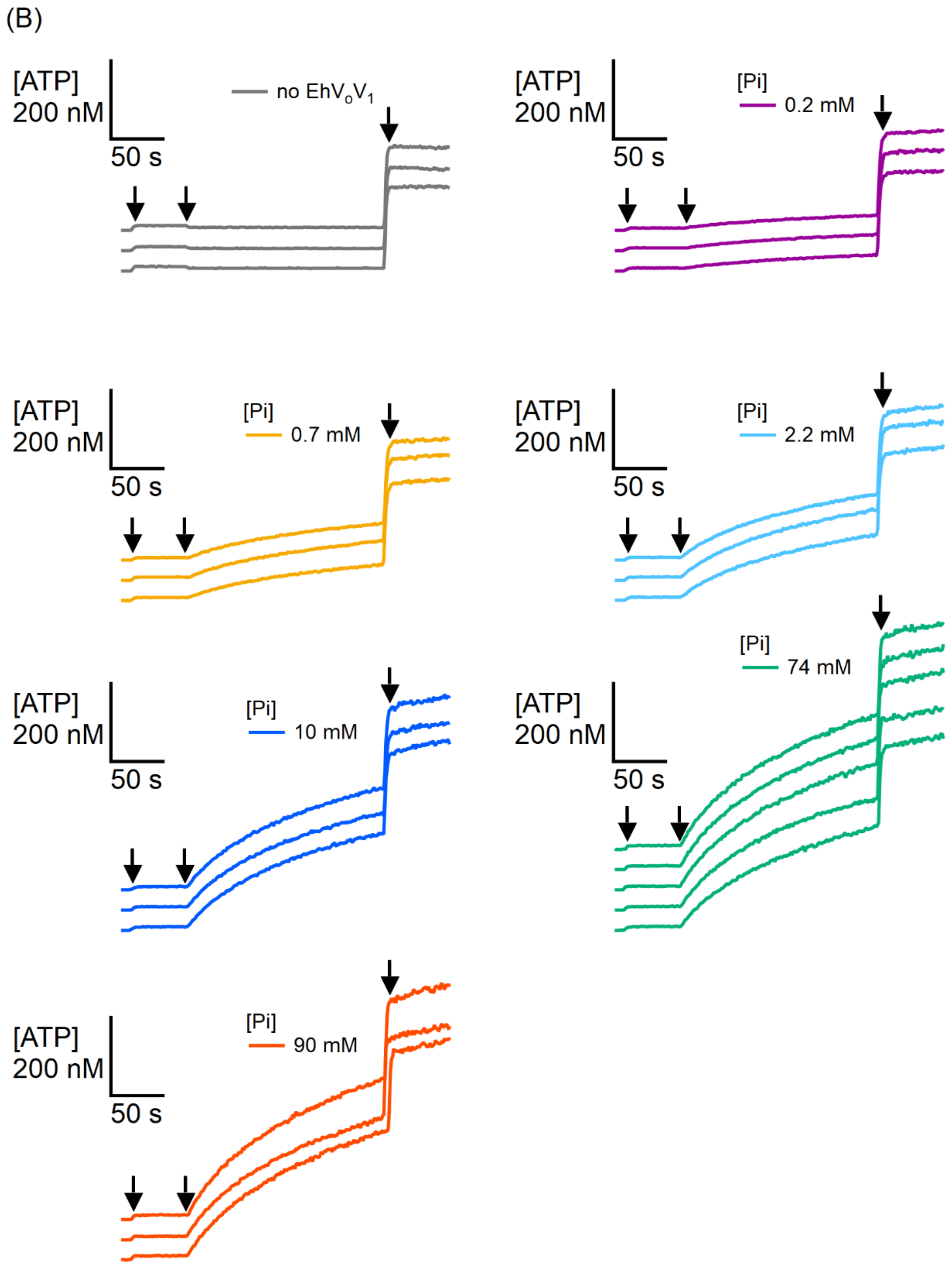


**Figure S3**. Time courses of [ADP] (A) and [Pi] (B) dependence of ATP synthesis activity of $EhV_oV_1$ under *smf* of 269.3 mV (Δψ of 154.7 mV and 2.3($k_BT/e$)·ΔpNa of 114.6 mV) shown in Figure 2A. ADP was varied (0.001 – 0.5 mM) with fixed [Pi] (74 mM) (A) and Pi was varied (0.2 – 90 mM) with fixed [ADP] (0.5 mM) (B). In both cases, proteoliposome (PL) or liposome without $EhV_oV_1$ for negative control experiments ([ADP] = 0.5 mM, [Pi] = 74 mM, gray traces), and ATP (final concentration: 200 nM) were added at 10, 60, and 240 sec, respectively, as indicated by black arrows.

(A)

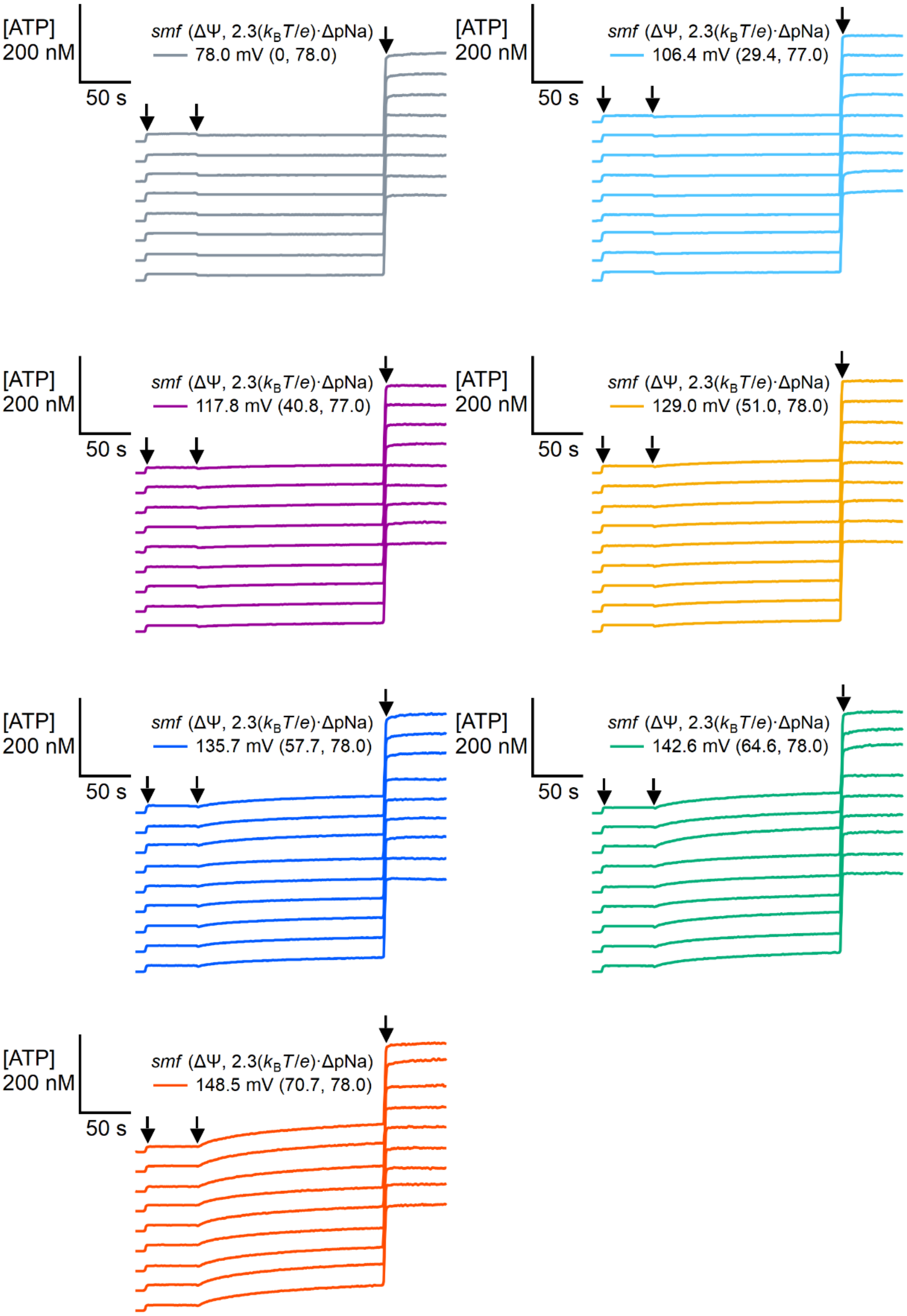
[ATP]
200 nM
50 s
smf (ΔΨ, 2.3(kBT/e)·ΔpNa)
78.0 mV (0, 78.0)
[ATP]
200 nM
50 s
smf (ΔΨ, 2.3(kBT/e)·ΔpNa)
106.4 mV (29.4, 77.0)
[ATP]
200 nM
50 s
smf (ΔΨ, 2.3(kBT/e)·ΔpNa)
117.8 mV (40.8, 77.0)
[ATP]
200 nM
50 s
smf (ΔΨ, 2.3(kBT/e)·ΔpNa)
129.0 mV (51.0, 78.0)
[ATP]
200 nM
50 s
smf (ΔΨ, 2.3(kBT/e)·ΔpNa)
135.7 mV (57.7, 78.0)
[ATP]
200 nM
50 s
smf (ΔΨ, 2.3(kBT/e)·ΔpNa)
142.6 mV (64.6, 78.0)
[ATP]
200 nM
50 s
smf (ΔΨ, 2.3(kBT/e)·ΔpNa)
148.5 mV (70.7, 78.0)

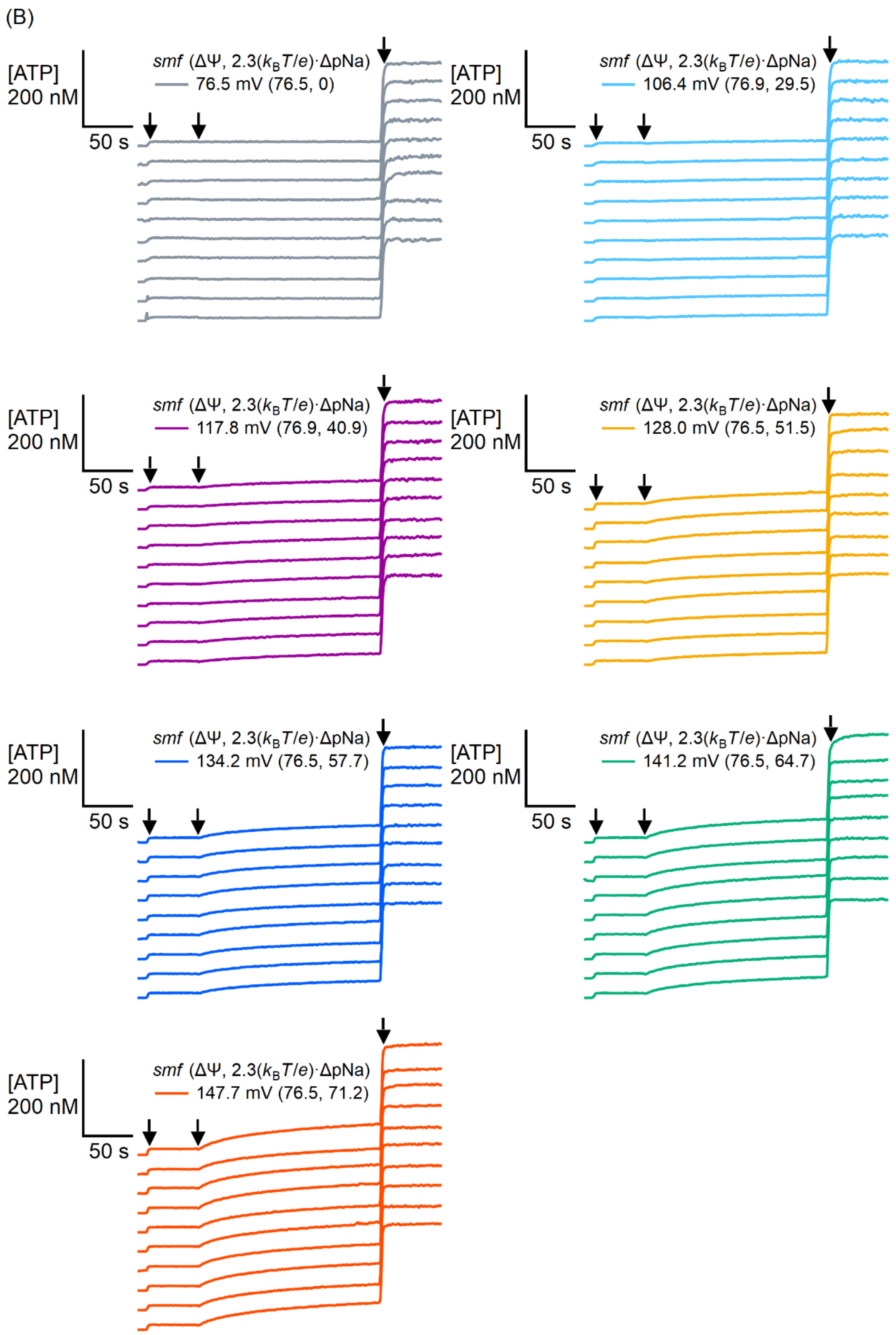


**Figure S4**. Time courses of ATP synthesis activity of $EhV_oV_1$ at different *smf* shown in Figure 3A ((A) and (B) correspond to the left and right panels, respectively). ADP (final concentration: 0.5 mM), PL, and ATP (final concentration: 200 nM) were added at 10, 60, and 240 sec, respectively.

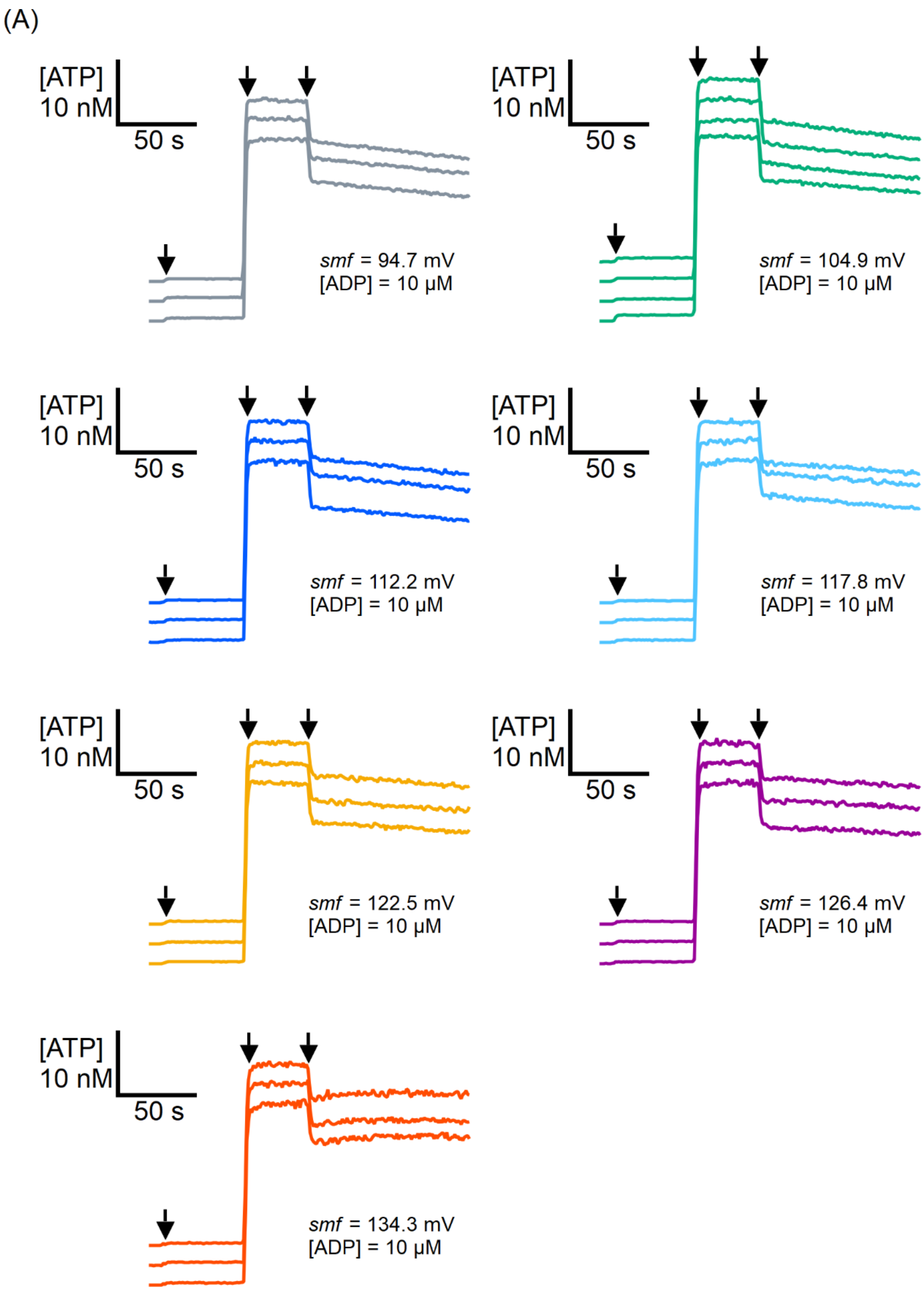
(A)
[ATP]
10 nM
50 s
smf = 94.7 mV
[ADP] = 10 μM
[ATP]
10 nM
50 s
smf = 104.9 mV
[ADP] = 10 μM
[ATP]
10 nM
50 s
smf = 112.2 mV
[ADP] = 10 μM
[ATP]
10 nM
50 s
smf = 117.8 mV
[ADP] = 10 μM
[ATP]
10 nM
50 s
smf = 122.5 mV
[ADP] = 10 μM
[ATP]
10 nM
50 s
smf = 126.4 mV
[ADP] = 10 μM
[ATP]
10 nM
50 s
smf = 134.3 mV
[ADP] = 10 μM

(B)

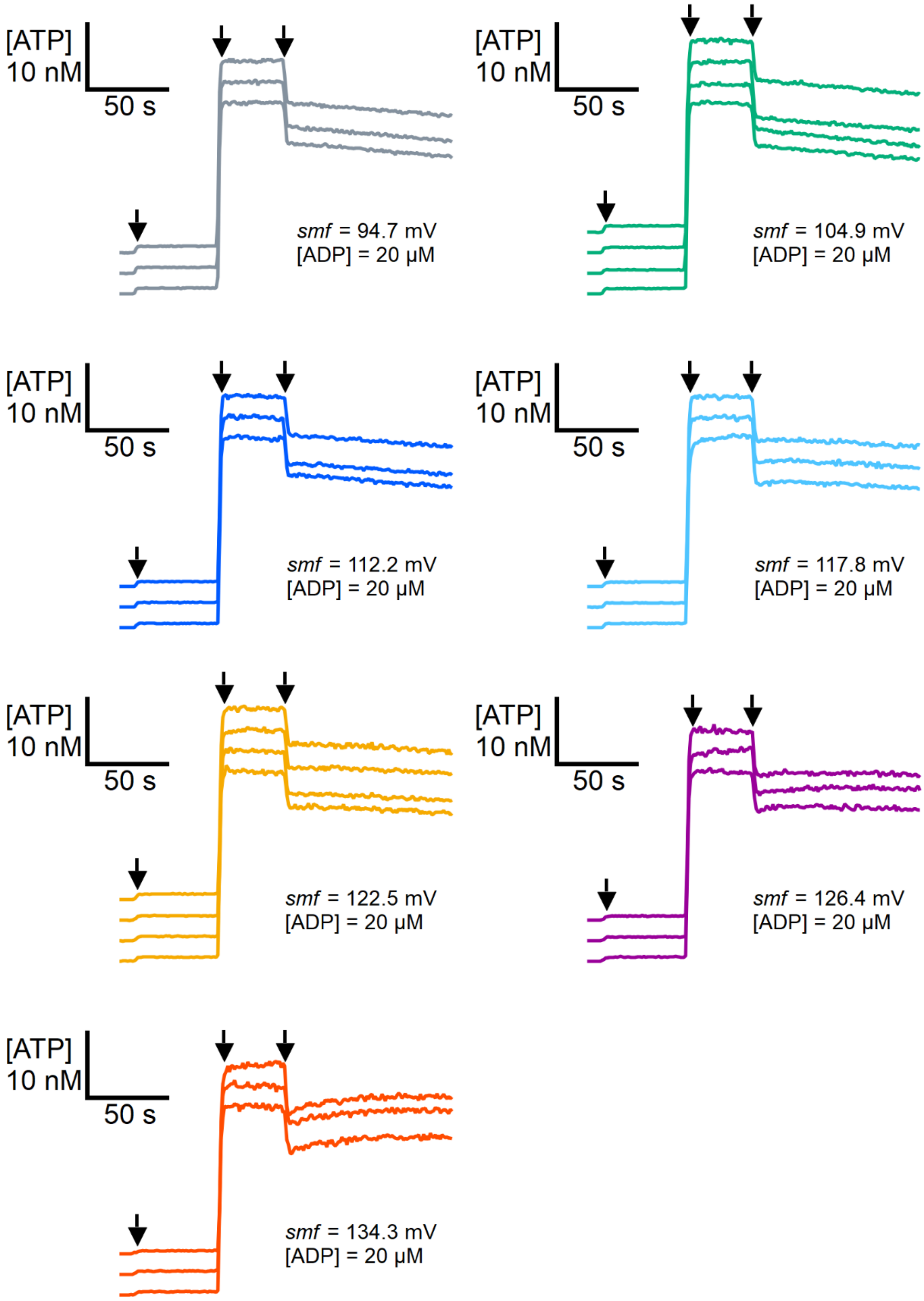
[ATP]
10 nM
50 s
smf = 94.7 mV
[ADP] = 20 μM
[ATP]
10 nM
50 s
smf = 104.9 mV
[ADP] = 20 μM
[ATP]
10 nM
50 s
smf = 112.2 mV
[ADP] = 20 μM
[ATP]
10 nM
50 s
smf = 117.8 mV
[ADP] = 20 μM
[ATP]
10 nM
50 s
smf = 122.5 mV
[ADP] = 20 μM
[ATP]
10 nM
50 s
smf = 126.4 mV
[ADP] = 20 μM
[ATP]
10 nM
50 s
smf = 134.3 mV
[ADP] = 20 μM

(C)

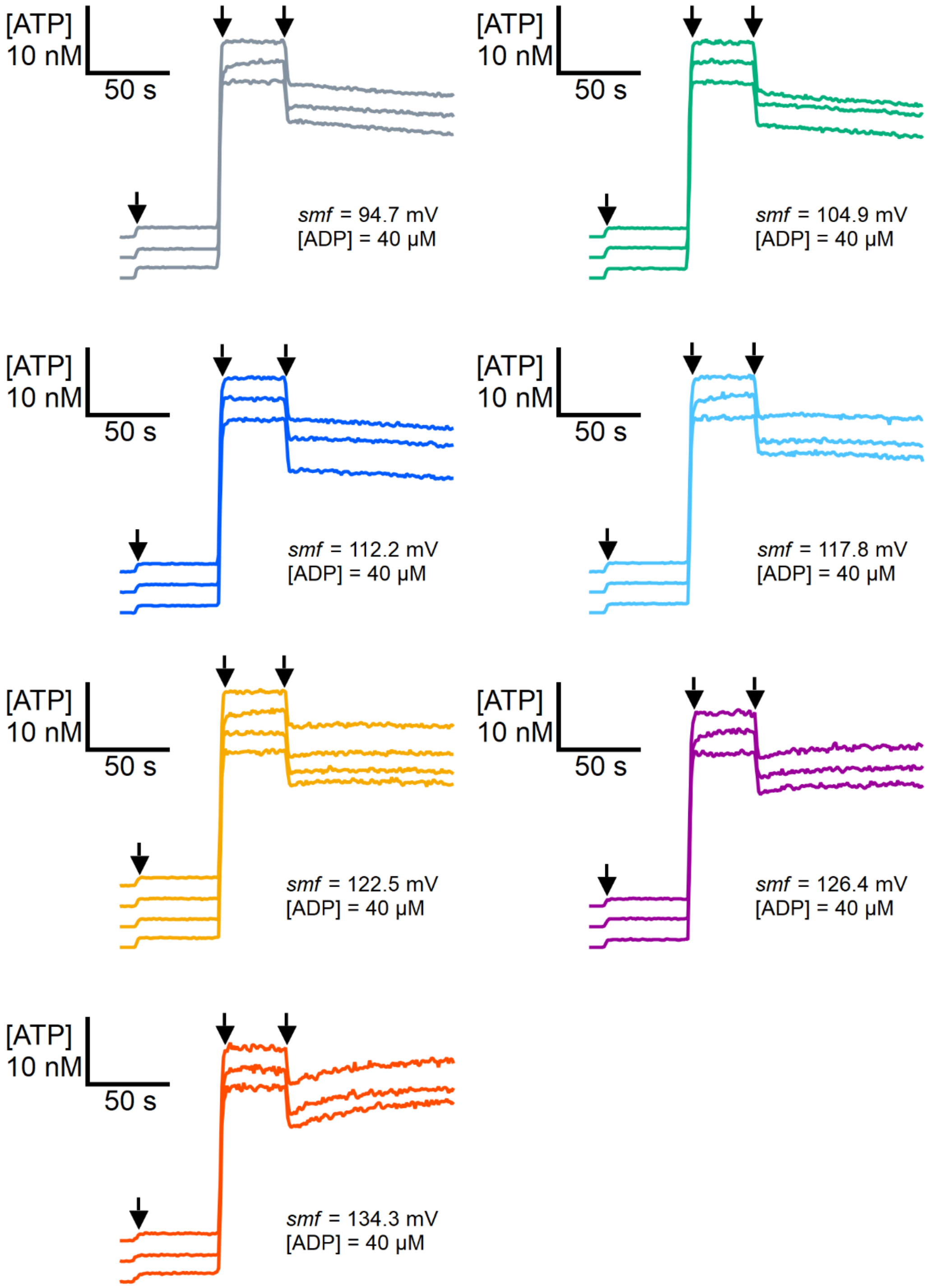
[ATP]
10 nM
50 s
smf = 94.7 mV
[ADP] = 40 μM
[ATP]
10 nM
50 s
smf = 104.9 mV
[ADP] = 40 μM
[ATP]
10 nM
50 s
smf = 112.2 mV
[ADP] = 40 μM
[ATP]
10 nM
50 s
smf = 117.8 mV
[ADP] = 40 μM
[ATP]
10 nM
50 s
smf = 122.5 mV
[ADP] = 40 μM
[ATP]
10 nM
50 s
smf = 126.4 mV
[ADP] = 40 μM
[ATP]
10 nM
50 s
smf = 134.3 mV
[ADP] = 40 μM

(D)

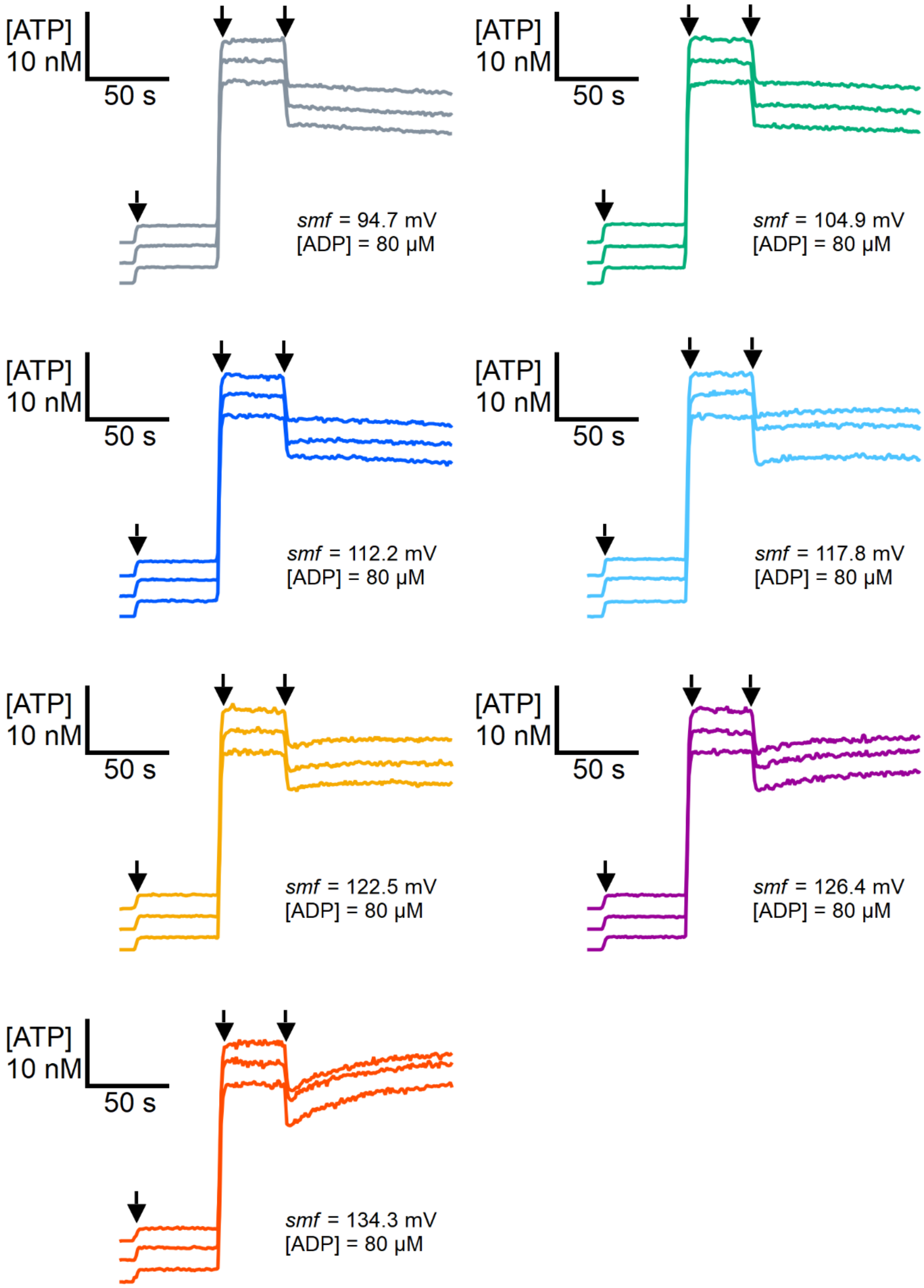


**Figure S5**. Time courses of ATP synthesis and hydrolysis activity of $EhV_oV_1$ at different *smf* (94.7 – 134.3 mV) corresponding to the panels A-D of Figure 4. ADP (10 - 80 µM), ATP (25 nM), and PL were added at 10, 60, and 100 sec, respectively, as indicated by black arrows. ADP concentrations were 10 µM (A), 20 µM (B), 40 µM (C), and 80 µM (D). Pi concentration was 9.95 mM for all conditions.
